# Prevalence and factors associated with tobacco use among men in India: findings from a nationally representative data

**DOI:** 10.1186/s12199-020-00898-x

**Published:** 2020-10-14

**Authors:** Md Shariful Islam, K. M. Saif-Ur-Rahman, Md. Mofijul Islam Bulbul, Deepak Singh

**Affiliations:** 1 Public Health Foundation, Bangladesh, Dhaka, Dhaka District Bangladesh; 2 Institute of Public Health Nutrition, Dhaka, Dhaka District Bangladesh; 3 icddr,b, Dhaka, Dhaka District Bangladesh; 4 National Nutrition Services, Dhaka, Dhaka District Bangladesh; 5 PATH, New Delhi, India

**Keywords:** Determinants, Factors, Prevalence, Tobacco consumption, Tobacco smoking, Smokeless tobacco, India

## Abstract

**Background:**

Tobacco consumption causes almost 638,000 premature deaths per year in India. This study sought to examine the prevalence and determinants of tobacco use among men in India.

**Methods:**

We analyzed data from the fourth round of the National Family Health Survey in India. These nationally representative cross-sectional sample data were collected from January 20, 2015, to December 4, 2016. A total of 112,122 men aged 15–54 years were included in this study. Primary outcomes were tobacco use categorized into smoking, smokeless, any tobacco, and both smoked and smokeless tobacco use. Complex survey design and sampling weights were applied in both the descriptive analyses and logistic regression models. We present the findings using odds ratios.

**Results:**

The prevalence of tobacco use among men in India for the studied period was 45.5% (95% CI 44.9–46.1), smoking was 24.6% (95% CI 24.1–25.1), smokeless tobacco use was 29.1% (95% CI 28.6–29.6), and both smoked and smokeless tobacco use was 8.4% (95% CI 8.1–8.7). The prevalence of tobacco use among men was higher among the elderly, separated/divorced/widowed individuals, those with lower education and wealth status, alcohol consumers, manual workers, and residents of the northeast region. Multivariate analysis showed that age, lower education, occupation, region, alcohol consumption, separated/divorced/widowed status, and economic status were substantially associated with tobacco use among Indian men.

**Conclusions:**

Innovative and cost-effective strategies targeting high-risk groups are crucial to curbing the tobacco epidemic in India. Anti-smoking campaigns should also focus on mitigating alcohol abuse. Reducing tobacco marketing and implementing formal education about the dangers of tobacco use, progressive taxing, packaging, and labeling of tobacco products and price strategies should be harmonized in legal provisions.

## Background

Tobacco consumption causes 8 million deaths every year worldwide [1]. Premature deaths attributable to tobacco are rising, an estimated 10 million deaths per year globally by 2030, while 70% of total deaths contributed by developing countries [2]. The chance of non-communicable diseases (NCDs), such as cardiovascular, respiratory diseases, and cancer, increased by smoking. Parental smoking is responsible for newborn death due to sudden infant death syndrome (SIDS), complications of low birth weight, prematurity, and other conditions [3, 4]. Along with the health burden, the economic loss attributable to smoking increased to a great extent which was equivalent to 1.8% of the world’s annual gross domestic product (GDP). The burden was substantially shared by developing countries [5].

The tobacco situation in India is more complex than any other country in the world with the use of a variety of smoking and smokeless tobacco products and a number of mixtures. Moreover, in India, many of these tobacco products are manufactured in cottage and small-scale industries [6]. In this second-largest tobacco-producing country, a total estimated 638,000 premature deaths per year occurred due to tobacco consumption [7]. India lost US$ 22.4 billion for all disease management caused by tobacco use in 2011 [8]. According to a recent study, the prevalence of tobacco use in India was 11.6%. Age, sex, education, wealth status, and alcohol consumers were associated with tobacco use in India [9]. Some other studies also measured the prevalence and factors of tobacco in India [10–13]. However, the evidence of tobacco consumption specifically among men in India is sparse and old. Some studies were conducted considering only one state with a limited scale [14, 15]. It is essential to understand the updated prevalence of tobacco use among men and its distribution and association between different population groups in this heterogeneous country to adjust and develop relevant health policy and interventions. The aims of this study are to measure the prevalence of tobacco use among men in India and the patterns of association of tobacco use with socio-demographic characteristics.

## Methodology

### Study design and population

We analyzed data originated from the fourth round of the National Family Health Survey (NFHS-4), a large-scale cross-sectional survey conducted in all 29 states and seven union territories in India [16]. Data were collected from January 20, 2015, to December 4, 2016. The NFHS-4 was performed using a 2-stage stratified survey designed with rural and urban stratification. In the first stage, a total of 28,586 primary sampling units (census enumeration blocks) listed in 2011 census data were identified. After listing all residential households, a fixed number of 22 households were selected in each primary sampling unit using systematic random sampling in the second stage. All women aged 15–49 years in the selected households were eligible to take part in the survey. In men’s survey, all men aged 15–54 who resided the night in a random subsample of 15% of these households were invited to participate. The household response rate was nearly 98%, and the individual response rate was 92% among eligible men. A total of 112,122 men aged 15–54 years were interviewed in the NFHS-4 who were recruited in this study. The details of the selection process are provided in the final report of NFHS-4 [16].

### Outcomes

The primary outcome of this study was “tobacco use”. The status of tobacco use of respondents measured by asking questions, such as does he use tobacco? What types of tobacco he does use? The primary outcome was further categorized into “any tobacco,” “smoking,” “smokeless tobacco (SLT),” and “both smoked and smokeless tobacco.” The respondent was classified under the “smoking” group when he reported smoking cigarettes or bidis (hand-rolled cigarettes) or cigar or pipe or hookah. If the men responded used chewing tobacco or snuff or gutkha/paan masala or paan with tobacco or khaini, he was categorized as an SLT group. Further, the participant was classified as any tobacco user if he used any type of tobacco and classified as both smoked and smokeless tobacco user if he used both smoked tobacco and SLT.

### Socio-demographic variables

We identified all the independent variables from the NFHS-4 survey related to men that we considered to be useful for fulfilling the aim of the study, which were identified based on the previously published studies and similar studies [9, 10, 12, 13]. Variables were categorized into four major levels—demographic, socio-economic, spatial, and access to information. Demographic factors included age of participants (15–24, 25–34, 35–44, and 45–54 years) and marital status (single, married, and separated/divorced/widow). Education (no education, primary, secondary, higher), occupation (not working, professional/technical/managerial/clerical/sale/services, agriculture, and skilled/unskilled manual), household’s wealth index (poorest, poorer, middle, richer, and richest), alcohol-consuming (yes or no), and ethnicity (caste, tribe, and no caste/tribe) were included in socio-economic factors. Spatial factors were the place of residence (urban and rural) and regions. We categorized all twenty-nine states and seven union territories into the six administrative regions, including south, west, north-east, east, central, and north [17]. The frequency of reading newspapers, watching television, and listening to the radio was grouped into access to information category.

### Statistical analyses

We conducted descriptive analyses using frequencies and proportions to quantify the distribution of the study population. We computed prevalence and proportion estimates with a 95% confidence interval. The prevalence was weighted using sampling weights mentioned in the dataset. The chi-squared test was conducted to explore the association of different forms of tobacco use with socio-demographic exposures. The association of the socio-demographic exposures and outcomes (smoking, SLT, any tobacco, and both smoked and SLT) was analyzed using logistic regression models to compute unadjusted and adjusted odds ratios (ORs) and 95% confidence interval (CI) and *p* value. Bivariate analysis was done between outcomes (smoking, SLT, any tobacco, and both smoked and SLT) and the socio-demographic exposures. We adjusted all selected socio-demographic exposures in multivariate regression model. We used survey analysis procedures in R statistical software version 4.0 accounting for the complex sampling design and sampling weights in all analyses described here, including these estimates and *p* value. *p* values of less than 0·05 were considered as significant.

### Ethical approval

This study is a secondary analysis based on the National Family Health Survey dataset which is available in the public domain. We applied for the NFHS-4 dataset stating the aims and objectives and granted approval.

## Results

### Socio-demographic characteristics of respondents

Of 112,122 men in India aged 15–54 years included in this study, 31.5% were from the 15–24 years age group (Table 1). Six out of ten men (63.1%) were married and 57.1% had completed secondary education. The highest proportion of the population was agriculture workers. The majority (89.6%) belonged to the caste ethnicity. Most of the respondents (81.5%) were from the Hindu religion. The proportion of the richest group (23.3%) is slightly higher than in other economic groups. In spatial factors, 61.7% of respondents lived in a rural area, and 23.9% were from southern regions (Table 1).

**Table 1:** Characteristics of the study population and prevalence of smoke and smokeless tobacco use among Indian men by socio-demographic characteristics, India 2015–2016^a^

Factors		Smoking		Smokeless	
	Total *N* (%)^b^	*n* (%)^b^	*p* value % (95% CI)	*n* (%)^b^	*p* value % (95% CI)
Overall	112,122 (100)	27,563 (100)	24.6 (24.1–25.1)	32,621 (100)	29.1 (28.6–29.6)
Demographic factors					
Age of the respondents			*P* < 0.001		*P* < 0.001
15–24	35,364 (31.5)	4570 (16.6)	12.9 (12.3–13.6)	6773 (20.8)	19.2 (18.5–19.8)
25–34	30,775 (27.5)	7742 (28.1)	25.2 (24.2–26.1)	10,373 (31.8)	33.7 (32.8–34.6)
35–44	25,821 (23.0)	7989 (29.0)	30.9 (30.0–31.9)	9075 (27.8)	35.1 (34.2–36.1)
45–54	20,162 (18.0)	7261 (26.3)	36.0 (35.0–37.1)	6401 (19.6)	31.7 (30.8–32.7)
Marital status			*P* < 0.001		*P* < 0.001
Single	39,763 (35.5)	5761 (20.9)	14.5 (13.8–15.2)	7272 (22.3)	18.3 (17.6–19.0)
Married	70,781 (63.1)	21,111 (76.6)	29.8 (29.2–30.5)	24,669 (75.6)	34.9 (34.2–35.5)
Separated/divorced/widow	1578 (1.4)	690 (2.5)	43.7 (39.7–47.8)	681 (2.1)	43.1 (39.2–47.2)
Sociocultural factors					
Education			*P* < 0.001		*P* < 0.001
No education	14,590 (13.0)	5922 (21.5)	40.6 (39.3–41.8)	6305 (19.3)	43.2 (41.9–44.6)
Primary	14,091 (12.6)	5068 (18.4)	36.0 (34.8–37.2)	5879 (18.0)	41.7 (40.4–43.0)
Secondary	64,010 (57.1)	13,646 (49.5)	21.3 (20.7–21.9)	17,798 (54.6)	27.8 (27.2–28.4)
Higher	19,431 (17.3)	2928 (10.6)	15.1 (14.0–16.1)	2639 (8.1)	13.6 (12.8–14.3)
Occupation			*P* < 0.001		*P* < 0.001
Not working	24,623 (22.0)	2941 (10.7)	11.9 (11.3–12.7)	3346 (10.3)	13.6 (13.0–14.2)
Professional/technical/managerial/services	28,191 (25.2)	6675 (24.3)	23.7 (22.7–24.7)	6655 (20.4)	23.6 (22.7–24.6)
Agriculture	30,202 (27.0)	8981 (32.6)	29.7 (28.9–30.6)	11,278 (34.6)	37.3 (36.5–38.2)
Skilled/unskilled manual	28 911 (25.8)	8907 (32.4)	30.8 (29.8–31.8)	11,290 (34.7)	39.0 (37.9–40.2)
Ethnicity			*p* < 0.001		*P* < 0.001
Caste	99,554 (89.6)	23,909 (87.7)	24.0 (23.5–24.6)	28,489 (88.3)	28.6 (28.1–29.2)
Tribe	7003 (6.3)	1860 (6.8)	26.6 (25.1–28.0)	2,884 (8.9)	41.2 (39.2–43.1)
No caste/tribe	4595 (4.1)	1490 (5.5)	32.4 (29.6–35.3)	905 (2.8)	19.7 (17.4–22.1)
Consuming alcohol			*p* < 0.001		*P* < 0.001
No	79036(70.5)	12281 (44.6)	15.5 (15.1–16.0)	18,597 (57.0)	23.5 (23.0–24.1)
Yes	33086(29.5)	15282 (55.4)	46.2 (45.1–47.2)	14,023 (43.0)	42.4 (41.4–43.4)
Religion			*p* < 0.001		*P* < 0.001
Hindu	91,390 (81.5)	22,278 (80.8)	24.4 (23.8–24.9)	27,428 (84.1)	30.0 (29.4–30.6)
Muslim	14,790 (13.2)	4083 (14.8)	27.6 (26.1–29.2)	4018(12.3)	27.2 (25.6–28.8)
Others	5942(5.3)	1202 (4.4)	20.2 (18.6–21.9)	1175(3.6)	19.8 (18.2–21.4)
Household’s wealth index			*P* < 0.001		*P* < 0.001
Poorest	16,441 (14.7)	5104 (18.5)	31.0 (30.0–32.1)	8045 (24.7)	48.9 (47.7–50.1)
Poorer	20,904 (18.6)	5992 (21.7)	28.7 (27.6–29.7)	7986 (24.5)	38.2 (37.2–39.2)
Middle	23,687 (21.1)	6011 (21.8)	25.4 (24.5–26.2)	6857 (21.0)	29.0 (28.0–29.9)
Richer	24,976 (22.3)	5669 (20.6)	22.7 (21.7–23.7)	5692 (17.4)	22.8 (21.9–23.7)
Richest	26,114 (23.3)	4787 (17.4)	18.3 (17.1–19.6)	4039 (12.4)	15.5 (14.5–16.5)
Spatial factors					
Place of residence			*P* < 0.001		*P* < 0.001
Rural	69,170 (61.7)	17,688 (64.1)	25.6 (25.0–26.1)	22,674 (69.5)	32.8 (32.2–33.3)
Urban	42,952 (38.3)	9875 (35.9)	23.0 (22.0–24.0)	9947(30.5)	23.2 (22.1–24.2)
Region			*P* < 0.001		*P* < 0.001
South	26,759 (23.9)	6708 (24.3)	25.1 (23.9–26.3)	2444 (7.5)	9.1 (8.5–9.8)
West	20,590 (18.3)	2709 (9.8)	13.2 (12.0–14.4)	7340 (22.5)	35.6 (33.9–37.4)
North-east	3693 (3.3)	1444 (5.2)	39.1 (37.3–40.9)	1729 (5.3)	46.8 (44.9–48.7)
East	21,051 (18.8)	5943 (21.6)	28.2 (26.9–29.6)	8166 (25.0)	8.8 (37.3–40.3)
Central	24,117 (21.5)	6434 (23.4)	26.7 (26.0–27.4)	10,341 (31.7)	42.9 (42.0–43.8)
North	15,912 (14.2)	4324 (15.7)	27.2 (26.3–28.1)	2600 (8.0)	16.3 (15.5–17.2)
Access to information					
Reading newspaper or magazine			*P* < 0.001		*P* < 0.001
Not at all	35,378 (31.6)	11,591 (42.1)	32.8 (31.9–33.6)	14085 (43.2)	39.8 (38.9–40.7)
Less than once a week	16,370 (14.6)	3852 (14.0)	23.5 (22.5–24.6)	5424 (16.6)	33.1 (31.9–34.3)
At least once a week	22,906 (20.4)	5026 (18.2)	21.9 (21.1–22.8)	6132 (18.8)	26.8 (25.9–27.7)
Almost every day	37,468 (33.4)	7093 (25.7)	18.9 (18.0–19.9)	6980 (21.4)	18.6 (17.9–19.4)
Frequency of watching television			*P* < 0.001		*P* < 0.001
Not at all	15,112 (13.5)	4289 (15.6)	28.4 (27.4–29.4)	6519 (20.0)	43.1 (41.9–44.3)
Less than once a week	10,419 (9.3)	2922 (10.6)	28.0 (26.9–29.2)	4400 (13.5)	42.2 (40.8–43.7)
At least once a week	16,911 (15.1)	4543 (16.5)	26.9 (25.8–27.9)	5836 (17.9)	34.5 (33.3–35.7)
Almost every day	69,680 (62.1)	15,808 (57.3)	22.7 (22.0–23.3)	15,866 (48.6)	22.8 (22.2–23.4)
Frequency of listening radio			*P* = 0.006		*P* < 0.001
Not at all	78,919 (70.4)	19,143 (69.5)	24.3 (23.7–24.8)	24,048 (73.7)	30.5 (29.8–31.1)
Less than once a week	9947 (8.9)	2668 (9.7)	26.8 (25.4–28.3)	3197 (9.8)	32.1 (30.6–33.7)
At least once a week	14,922 (13.3)	3782 (13.7)	25.3(23.9–26.8)	3696 (11.3)	24.8 (23.4–26.1)
Almost every day	8334 (7.4)	1969 (7.1)	23.6(22.1–25.2)	1679 (5.1)	20.1 (18.7–21.6)

^a^Data are from the fourth Indian National Family Health Survey. Frequency and prevalence with 95% CIs in parentheses are shown. All data are weighted to account for survey design. *P* value was calculated through the chi-square test

^b^Column percentages

### Prevalence of different forms of tobacco use

**Fig. 1:**
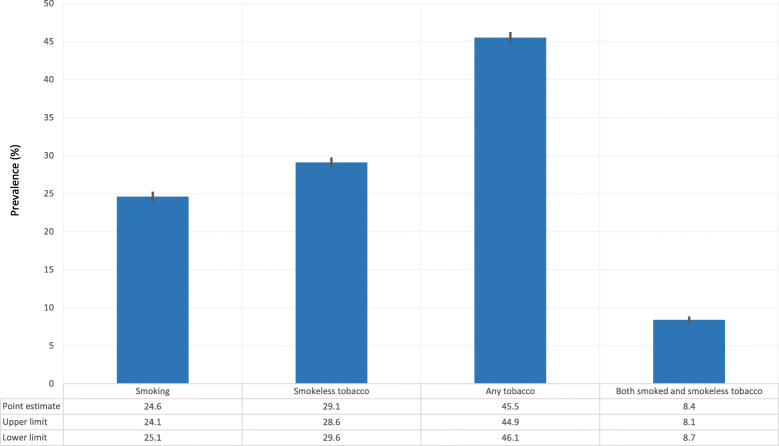
Prevalence of tobacco use among men in India, NFHS-4, 2015–2016

The overall prevalence of any forms of tobacco use among men in India was 45.5% (95% CI 44.9–46.1). The prevalence of smoking and smokeless tobacco was 24.6% (95% CI 24.1–25.1) and 29.1% (95% CI 28.6–29.6) respectively while 8.4% (95% CI 8.1-8.7) men in India used both smoked and smokeless tobacco (Fig. 1).

Tables 1 and 2 list the weighted estimates of the prevalence with 95% CI, *p* value, and frequency of tobacco use by socio-demographic characteristics. The prevalence of tobacco use increased with age. Similarly, separated/divorced/widow men used tobacco significantly higher than single and married men. However, the proportion of using tobacco decreased with a higher education level. The prevalence of tobacco use was higher among manual workers, men with poorest economic status, and who were from the rural and north-east regions. A substantially greater proportion of men who consumed alcohol used any tobacco (70.9%; 95% CI 69.9–71.9; *p* < 0.001). Muslim men smoked higher, whereas men from the Hindu religion used smokeless tobacco more. The respondents from the tribe ethnicity used smokeless, any forms of tobacco, and both smoked and smokeless tobacco higher than the other two ethnic groups; however, men who have no caste/tribe used smoked tobacco more.

**Table 2:** Characteristics of the study population and prevalence of any tobacco and both smoked and smokeless tobacco use among Indian men by socio-demographic characteristics, India 2015–2016^a ^

Factors		Any tobacco		Both smoked and smokeless	
	Total ***N*** (%)^**b**^	*n* (%)^b^	*p* value % (95% CI)	*n* (%)^b^	*p* value % (95% CI)
Overall	112,122 (100)	51,039 (100)	45.5 (44.9–46.1)	9398 (100)	8.4 (8.1–8.7)
Demographic factors					
Age of the respondents			*P* < 0.001		*P* < 0.001
15–24	35,364 (31.5)	9656 (18.9)	27.3 (26.5–28.1)	1731 (18.4)	4.9 (4.6–5.2)
25–34	30,775 (27.5)	15,269 (29.9)	49.6 (48.6–50.7)	2922 (31.1)	9.5 (9.0–10.0)
35–44	25,821 (23.0)	14,486 (28.4)	56.1 (55.1–57.1)	2653 (28.2)	10.3 (9.8–10.8)
45–54	20,162 (18.0)	11,628 (22.8)	57.7 (56.6–58.8)	2092 (22.3)	10.4 (9.8–11.0)
Marital status			*P* < 0.001		*P* < 0.001
Single	39,763 (35.5)	11,092 (21.7)	27.9 (27.1–28.7)	1993 (21.2)	5.0 (4.7–5.4)
Married	70781 (63.1)	38,843 (76.1)	54.9 (54.2–55.6)	7134 (75.9)	10.1 (9.7–10.4)
Separated/divorced/widowed	1578 (1.4)	1104 (2.2)	70.0 (66.2–73.5)	272 (2.9)	17.2 (14.5–20.1)
Sociocultural factors					
Education			*P* < 0.001		*P* < 0.001
No education	14,590 (13.0)	9985 (19.6)	68.4 (67.2–69.7)	2275 (24.2)	15.6 (14.7–16.5)
Primary	14,091 (12.6)	9145 (17.9)	64.9 (63.7–66.1)	1861 (19.8)	13.2 (12.5–14.0)
Secondary	64,010 (57.1)	26,934 (52.8)	42.1 (41.4–42.7)	4651 (49.5)	7.3 (7.0–7.6)
Higher	19,431 (17.3)	4975 (9.7)	25.6 (24.4–26.8)	611 (6.5)	3.1 (2.8–3.5)
Occupation			*P* < 0.001		*P* < 0.001
Not working	24,623 (22.0)	5449 (10.7)	22.1 (21.3–23.0)	881 (9.4)	3.6 (3.3–3.9)
Professional/technical/managerial/services	28,191 (25.2)	11,454 (22.5)	40.6 (39.5–41.8)	1910 (20.4)	6.8 (6.3–7.3)
Agriculture	30,202 (27.0)	17,144 (33.6)	56.8 (55.9–57.6)	3208 (34.1)	10.6 (10.1–11.1)
Skilled/unskilled manual	28,911 (25.8)	16,895 (33.2)	58.4 (57.4–59.5)	3384 (36.1)	11.7 (11.1–12.3)
Ethnicity			*P* < 0.001		*P* < 0.001
Caste	99,554 (89.6)	44,383 (87.9)	44.6 (44.0–45.2)	8198 (88.3)	8.2 (8.0–8.5)
Tribe	7003 (6.3)	4010 (7.9)	57.3 (55.4–59.1)	795 (8.6)	11.4 (10.4–12.3)
No caste/tribe	4595 (4.1)	2116 (4.2)	46.0 (43.2–48.9)	287 (3.1)	6.2 (5.1–7.5)
Consuming alcohol			*P* < 0.001		*P* < 0.001
No	79,036 (70.5)	27,582 (54.0)	34.9 (34.3–35.5)	3433 (36.5)	4.3 (4.1–4.6)
Yes	33,086 (29.5)	23,457 (46.0)	70.9 (69.9–71.9)	5965 (63.5)	18.0 (17.4–18.7)
Religion			*P* < 0.001		*P* = 0.075
Hindu	91,390 (81.5)	42,115 (82.5)	46.1 (45.4–46.7)	7831 (83.3)	8.6 (8.3–8.9)
Muslim	14,790 (13.2)	6903 (13.5)	46.7 (44.8–48.6)	1207 (12.9)	8.2 (7.5–8.9)
Others	5942 (5.3)	2021 (4.0)	34.0 (32.2–35.9)	359 (3.8)	6.0 (5.3–6.9)
Household’s wealth index			*P* < 0.001		*P* < 0.001
Poorest	16,441 (14.7)	10,552 (20.7)	64.2 (63.1–65.3)	2671 (28.4)	16.2 (15.5–17.0)
Poorer	20,904 (18.6)	11,693 (22.9)	55.9 (54.8–57.0)	2369 (25.2)	11.3 (10.7–11.9)
Middle	23,687 (21.1)	11,038 (21.6)	46.6 (45.6–47.6)	1883 (20.0)	8.0 (7.5–8.4)
Richer	24,976 (22.3)	9923 (19.4)	39.7 (38.6–40.8)	1467 (15.6)	5.9 (5.4–6.3)
Spatial factors					
Place of residence			*P* < 0.001		*P* < 0.001
Rural	69,170 (61.7)	34,004 (66.6)	49.2 (48.6–49.8)	6579 (70.0)	9.5 (9.2–9.8)
Urban	42,952 (38.3)	17,035 (33.4)	39.7 (38.5–40.8)	2820 (30.0)	6.6 (6.1–7.1)
Region			*P* < 0.001		*P* < 0.001
South	26,759 (23.9)	8332 (16.3)	31.1 (29.9–32.4)	830 (8.8)	3.1 (2.7–3.5)
West	20,590 (18.3)	8997 (17.6)	43.7 (42.0–45.4)	1070 (11.4)	5.2 (4.5–6.0)
North-east	3693 (3.3)	2431 (4.8)	65.8 (63.9–67.7)	755 (8.0)	20.4 (19.1–21.9)
East	21,051 (18.8)	11,675 (22.9)	55.5 (54.0–56.9)	2462 (26.2)	11.7 (10.9–12.5)
Central	24,117 (21.5)	13,546 (26.5)	56.2 (55.3–57.0)	3413 (36.3)	14.2 (13.6–14.7)
North	15,912 (14.2)	6057 (11.9)	38.1 (37.1–39.1)	869 (9.3)	5.5 (5.0–6.0)
Access to information					
Reading newspaper or magazine			*P* < 0.001		*P* < 0.001
Not at all	35,378 (31.6)	21,339 (41.8)	60.3 (59.4–61.2)	4441 (47.3)	12.6 (12.0–13.1)
Less than once a week	16,370 (14.6)	7835 (15.4)	47.9 (46.5–49.2)	1497 (15.9)	9.1 (8.4–9.9)
At least once a week	22,906 (20.4)	9519 (18.6)	41.6 (40.5–42.6)	1687 (17.9)	7.4 (6.9–7.8)
Almost every day	37,468 (33.4)	12,346 (24.2)	33.0 (31.9–34.0)	1772 (18.9)	4.7 (4.4–5.1)
Frequency of watching television			*P* < 0.001		*P* < 0.001
Not at all	15,112 (13.5)	8909 (17.4)	59.0 (57.8–60.1)	1934 (20.6)	12.8 (12.1–13.5)
Less than once a week	10,419 (9.3)	5954 (11.7)	57.1 (55.7–58.6)	1396 (14.9)	13.4 (12.5–14.3)
At least once a week	16,911 (15.1)	8659 (17.0)	51.2 (50.0–52.4)	1781 (18.9)	10.5 (9.9–11.2)
Almost every day	69,680 (62.1)	27,518 (53.9)	39.5 (38.7–40.2)	4288 (45.6)	6.2 (5.8–6.5)
Frequency of listening radio			*P* < 0.001		*P* < 0.001
Not at all	78,919 (70.4)	36,832 (72.2)	46.7 (46.0–47.3)	6556 (69.8)	8.3 (8.0–8.6)
Less than once a week	9947 (8.9)	4816 (9.4)	48.4 (46.7–50.1)	1068 (11.4)	10.7 (9.9–11.7)
At least once a week	14,922 (13.3)	6287 (12.3)	42.1 (40.6–43.7)	1217 (12.9)	8.2 (7.4–8.9)
Almost every day	8334 (7.4)	3104 (6.1)	37.2 (35.4–39.1)	557 (5.9)	6.7 (5.9–7.6)

^a^Data are from the fourth Indian National Family Health Survey. Frequency and prevalence with 95% CIs in parentheses are shown. All data are weighted to account for survey design. *P* value was calculated through the chi-square test

^b^Column percentages

### Factors associated with smoking and smokeless tobacco use

Table 3 presents the finding of an unadjusted and adjusted model showing the association of factors with smoking and smokeless tobacco (SLT) use among Indian men. The strength of the association of smoking increased with age while the adjusted odds ratio of using SLT decreased with age. The adjusted odds ratio of smoking was 2.46 (95% CI 2.21–2.74) among 45–54 years old men, and SLT use had an AOR (adjusted odds ratio) of 1.43 (95% CI 1.33–1.55) among men aged 25–34 years. Education level had a protective effect on smoking and SLT use; however, the association of primary level education became insignificant in the adjusted model. Manual work correlated to smoking (AOR = 1.55; 95% CI 1.40–1.72) and SLT use (AOR 2.25; 95% CI 2.07–2.45). Both unadjusted and adjusted models showed consuming alcohol associated with smoking and SLT use. The men in households with richest economic status were less likely being SLT user (unadjusted odds ratio = 0.191; 95% CI 0.175–0.209 and AOR = 0.437; 95% CI 0.386–0.495). The magnitude of the north-east region was stronger for using smoking and SLT in both bivariate and multivariate models. Separated/divorced/widow, no caste/tribe, Muslim, urban residents were positively associated with smoking, whereas, tribe ethnicity and other religions had a protective effect on smoking. Respondents from no caste/tribe and other religious groups had lower odds of SLT consuming.

**Table 3:** Logistic regression to identify factors associated with smoking and smokeless tobacco use among men, India 2015–2016^a^

Factors	Smoking				Smokeless			
	Unadjusted		Adjusted		Unadjusted		Adjusted	
	OR (95% CI)	*P* value	OR (95% CI)	*P* value	OR (95% CI)	*P* value	OR (95% CI)	*P* value
**Demographic factors**								
Age of the respondents								
15–24	1 (ref)		1 (ref)		1 (ref)		1 (ref)	
25–34	2.27 (2.11–2.43)	< 0.001	1.50 (1.36–1.65)	< 0.001	2.15 (2.04–2.26)	< 0.001	1.43 (1.33–1.55)	< 0.001
35–44	3.02 (2.82–3.23)	< 0.001	1.87 (1.69–2.06)	< 0.001	2.29 (2.16–2.43)	< 0.001	1.34 (1.23–1.47)	< 0.001
45–54	3.79 (3.55–4.05)	< 0.001	2.46 (2.21–2.74)	< 0.001	1.96 (1.85–2.08)	< 0.001	1.19 (1.08–1.30)	< 0.001
Marital status								
Single	1 (ref)		1 (ref)		1 (ref)		1 (ref)	
Married	2.51 (2.37–2.66)	< 0.001	1.03 (0.939–1.14)	0.496	2.39 (2.28–2.50)	< 0.001	1.44 (1.33–1.56)	< 0.001
Separated/divorced/widowed	4.59 (3.87–5.43)	< 0.001	1.70 (1.38–2.10)	< 0.001	3.39 (2.86–4.01)	< 0.001	1.60 (1.25–2.05)	< 0.001
**Sociocultural factors**								
Education								
No education	1 (ref)		1 (ref)		1 (ref)		1 (ref)	
Primary	0.822 (0.766–0.882)	< 0.001	0.929 (0.856–1.01)	0.078	0.941 (0.877-1.01)	0.09	1.03 (0.945–1.12)	0.527
Secondary	0.397 (0.374–0.421)	< 0.001	0.650 (0.597–0.708)	< 0.001	0.506 (0.476-0.538)	< 0.001	0.880 (0.806–0.960)	0.004
Higher	0.260 (0.235–0.287)	< 0.001	0.512 (0.453–0.579)	< 0.001	0.207 (0.190-0.225)	< 0.001	0.530 (0.470–0.599)	< 0.001
Occupation								
Not working	1 (ref)		1 (ref)		1 (ref)		1 (ref)	
Professional/technical/managerial/services	2.29 (2.10–2.49)	< 0.001	1.38 (1.25-1.52)	< 0.001	1.96 (1.83–2.11)	<0.001	1.62 (1.49-1.76)	< 0.001
Agriculture	3.12 (2.89–3.37)	< 0.001	1.52 (1.39-1.68)	< 0.001	3.79 (3.55–4.04)	< 0.001	1.77 (1.63–1.91)	< 0.001
Skilled/unskilled manual	3.28 (3.02–3.57)	< 0.001	1.55 (1.40–1.72)	< 0.001	4.07 (3.80–4.37)	< 0.001	2.25 (2.07–2.45)	< 0.001
Ethnicity								
Caste	1 (ref)		1 (ref)		1 (ref)		1 (ref)	
Tribe	1.14 (1.06–1.24)	< 0.001	0.830 (0.758–0.910)	< 0.001	1.75 (1.60–1.90)	< 0.001	0.977 (0.893–1.07)	0.605
No caste/tribe	1.52 (1.33–1.73)	< 0.001	1.42 (1.23–1.64)	< 0.001	0.611 (0.526–0.711)	< 0.001	0.682 (0.561–0.829)	< 0.001
Alcohol consuming								
No	1 (ref)		1 (ref)		1 (ref)		1 (ref)	
Yes	4.67 (4.42–4.93)	< 0.001	4.33 (4.08–4.60)	< 0.001	2.39 (2.28–2.51)	< 0.001	2.71 (2.56–2.86)	< 0.001
Religion								
Hindu	1 (ref)		1 (ref)		1 (ref)		1 (ref)	
Muslim	1.18 (1.09–1.29)	< 0.001	1.51 (1.38–1.66)	< 0.001	0.870 (0.799–0.947)	0.001	1.02 (0.930–1.12)	0.649
Others	0.786 (0.707–0.874)	0.342	0.666 (0.589–0.754)	< 0.001	0.575 (0.519–0.637)	< 0.001	0.635 (0.569–0.709)	< 0.001
Household’s wealth index								
Poorest	1 (ref)		1 (ref)		1 (ref)		1 (ref)	
Poorer	0.892 (0.837–0.952)	< 0.001	1.09 (1.01–1.18)	0.021	0.645 (0.608–0.684)	< 0.001	0.885 (0.826–0.948)	< 0.001
Middle	0.755 (0.709–0.805)	< 0.001	0.999 (0.913–1.09)	0.973	0.425 (0.399–0.453)	< 0.001	0.764 (0.708–0.825)	< 0.001
Richer	0.652 (0.605–0.703)	< 0.001	0.921 (0.832–1.02)	0.111	0.308 (0.287–0.330)	< 0.001	0.631 (0.576–0.692)	< 0.001
Richest	0.499 (0.452–0.550)	< 0.001	0.786 (0.690–0.896)	< 0.001	0.191 (0.175–0.209)	< 0.001	0.437 (0.386–0.495)	< 0.001
**Spatial factors**								
Place of residence								
Rural	1 (ref)		1 (ref)		1 (ref)		1 (ref)	
Urban	0.869 (0.82–0.93)	< 0.001	1.13 (1.05–1.22)	< 0.001	0.62 (0.578–0.668)	< 0.001	1.04 (0.970–1.18)	0.273
Region								
South	1 (ref)		1 (ref)		1 (ref)		1 (ref)	
West	0.453 (0.400–0.513)	< 0.001	0.673 (0.591–0.766)	< 0.001	5.51 (4.94–6.15)	< 0.001	8.40 (7.51–9.40)	< 0.001
North-east	1.92 (1.74–2.12)	< 0.001	1.98 (1.76–2.23)	< 0.001	8.76 (7.84–9.79)	< 0.001	9.38 (8.19–10.7)	< 0.001
East	1.18 (1.07–1.29)	< 0.001	1.26 (1.13–1.41)	< 0.001	6.31 (5.69–6.98)	< 0.001	6.18 (5.50–6.94)	< 0.001
Central	1.09 (1.01–1.17)	0.028	1.38 (1.27–1.50)	< 0.001	7.47 (6.84–8.15)	< 0.001	8.52 (7.74–9.39)	< 0.001
North	1.12 (1.03–1.21)	0.007	1.73 (1.58–1.90)	< 0.001	1.94 (1.76–2.15))	< 0.001	2.62 (2.35–2.92)	< 0.001
**Access to information**								
Reading newspaper or magazine								
Not at all	1 (ref)		1 (ref)		1 (ref)		1 (ref)	
Less than once a week	0.632 (0.589–0.677)	< 0.001	0.950 (0.872–1.04)	0.249	0.749 (0.702–0.799)	< 0.001	0.945 (0.874–1.02)	0.155
At least once a week	0.577 (0.543–0.613)	< 0.001	0.946 (0.877–1.02)	0.150	0.553 (0.522–0.585)	< 0.001	0.924 (0.857–0.997)	0.042
Almost every day	0.479 (0.447–0.513)	< 0.001	0.865 (0.794–0.942)	< 0.001	0.346 (0.326–0.368)	< 0.001	0.875 (0.804–0.953)	0.002
Frequency of watching television								
Not at all	1 (ref)		1 (ref)		1 (ref)		1 (ref)	
Less than once a week	0.984 (0.912–1.06)	0.666	1.10 (1.01–1.20)	0.032	0.964 (0.895–1.04)	0.33	1.05 (0.962–1.15)	0.272
At least once a week	0.927 (0.863–0.996)	0.039	1.13 (1.03–1.23)	0.007	0.695 (0.649–0.744)	< 0.001	0.982 (0.906–1.06)	0.660
Almost every day	0.740 (0.695–0.788)	< 0.001	1.14 (1.04–1.25)	0.005	0.389 (0.366–0.412)	< 0.001	0.924 (0.854–1.00)	0.052
Frequency of listening radio								
Not at all	1 (ref)		1 (ref)		1 (ref)		1 (ref)	
Less than once a week	1.14 (1.06–1.24)	< 0.001	1.16 (1.06–1.26)	0.001	1.08 (1.00–1.17)	0.047	1.06 (0.970–1.16)	0.194
At least once a week	1.06 (0.979–1.15)	0.15	1.13 (1.03–1.23)	0.009	0.751 (0.695–0.812)	< 0.001	0.892 (0.821–0.969)	0.006
Almost every day	0.966 (0.880–1.06)	0.47	1.12 (1.02–1.24)	0.021	0.576 (0.523–0.633)	< 0.001	0.886 (0.796–0.986)	0.026

^a^Data are from the fourth Indian National Family Health Survey. Unadjusted and adjusted odds ratios with 95% CIs in parentheses are shown. *P* value was displayed in a separate column. All data are weighted to account for survey design

In access to information, watching television and listening to radio significantly correlated with smoking in the adjusted model; however, the magnitude was much weaker. The association of reading newspapers or magazines at less than or at least once a week with smoking became insignificant in the adjusted model. Reading newspapers and listening to the radio at least once a week and almost every day had a protective impact on SLT use while the association of watching television with SLT use was insignificant in the multivariate regression model.

### Factors associated with any tobacco and both smoked and smokeless tobacco use

Table 4 shows the findings of bivariate and multivariate regression of the factors associated with any forms of tobacco use and the dual use of smoked and SLT among Indian men. Age was positively associated with using any forms of tobacco, and the direction continued steadily in the adjusted model. Men aged 45–54 years had around two times odds (AOR = 2.14; 95% CI 1.96–2.35) than men aged 15–24 years old. Compared with single men, the individuals who were separated/divorced/widow had higher odds (AOR = 2.13; 95% CI 1.68–2.70) of being a tobacco user. Education level has inversely correlated with tobacco use among Indian men. The adjusted odds were 0.444 (95% CI 0.398–0.496) among men with higher education. The respondents who worked in any occupations had a higher odds ratio than the men who did not work. The odds ratio of using tobacco was 4.54 (95% CI 4.30–4.80) among alcohol consumers in the unadjusted model; however, the ratio (AOR = 5.22; 95% CI 4.88–5.59) was greater in the adjusted model. Household wealth status was negatively associated with any forms of tobacco use. The odds ratio of using tobacco in the highest quintile of household wealth was 0.482 (95% CI 0.435–0.534) in the multivariate model. Respondents from the north-east region were more likely (AOR = 5.22, 95% CI 4.57–5.98) to be a tobacco user. Reading the newspaper, at least once a week and almost every day associated with tobacco use in the adjusted model, whereas, the association with watching television or listening radio was insignificant. The association of using tobacco use with different ethnic groups, religions, and living in urban areas was found insignificant in our multivariate regression analysis.

**Table 4:** Logistic regression to identify factors associated with any tobacco and both smoked and smokeless tobacco use among men, India 2015–2016^a^

Factors	Any tobacco				Both smoke and smokeless tobacco			
	Unadjusted		Adjusted		Unadjusted		Adjusted	
	OR (95% CI)	*P* value	OR (95% CI)	*P* value	OR (95% CI)	*P* value	OR (95% CI)	*P* value
**Demographic factors**								
Age of the respondents								
15–24	1 (ref)		1 (ref)		1 (ref)		1 (ref)	
25–34	2.62 (2.48–2.77)	< 0.001	1.65 (1.53–1.78)	< 0.001	2.04 (1.88–2.21)	< 0.001	1.35 (1.21–1.50)	< 0.001
35–44	3.40 (3.22–3.59)	< 0.001	1.90 (1.74–2.08)	< 0.001	2.22 (2.04–2.43)	< 0.001	1.34 (1.18–1.52)	< 0.001
45–54	3.63 (3.43–3.83)	< 0.001	2.14 (1.96–2.35)	< 0.001	2.25 (2.05–2.47)	< 0.001	1.44 (1.26–1.64)	< 0.001
Marital status								
Single	1 (ref)		1 (ref)		1 (ref)		1 (ref)	
Married	3.14 (3.01–3.28)	< 0.001	1.36 (1.26–1.46)	< 0.001	2.12 (1.96–2.30)	< 0.001	1.06 (0.945–1.20)	0.3118
Separated/divorced/widowed	6.02 (5.04–7.19)	< 0.001	2.13 (1.68–2.70)	< 0.001	3.94 (3.20–4.84)	< 0.001	1.56 (1.22–1.98)	< 0.001
**Sociocultural factors**								
Education								
No education	1 (ref)		1 (ref)		1 (ref)		1 (ref)	
Primary	0.853 (0.792–0.918)	< 0.001	0.991 (0.915–1.07)	0.830	0.824 (0.754–0.899)	< 0.001	0.919 (0.830–1.02)	0.104
Secondary	0.335 (0.315–0.356)	< 0.001	0.673 (0.620–0.731)	< 0.001	0.424 (0.391–0.460)	< 0.001	0.737 (0.659–0.824)	< 0.001
Higher	0.159 (0.146–0.173)	< 0.001	0.444 (0.398–0.496)	< 0.001	0.176 (0.153–0.202)	< 0.001	0.450 (0.375–0.540)	< 0.001
Occupation								
Not working	1 (ref)		1 (ref)		1 (ref)		1 (ref)	
Professional/technical/managerial/services	2.41 (2.25–2.57)	<0.001	1.54 (1.42–1.67)	< 0.001	1.96 (1.75–2.19)	< 0.001	1.46 (1.28–1.66)	< 0.001
Agriculture	4.62 (4.35–4.91)	< 0.001	1.81 (1.67–1.95)	< 0.001	3.20 (2.87–3.57)	< 0.001	1.47 (1.30–1.66)	< 0.001
Skilled/unskilled manual	4.95 (4.63–5.29)	< 0.001	2.21 (2.04–2.40)	< 0.001	3.57 (3.20–3.98)	< 0.001	1.67 (1.47–1.90)	< 0.001
Ethnicity								
Caste	1 (ref)		1 (ref)		1 (ref)		1 (ref)	
Tribe	1.67 (1.54–1.80)	< 0.001	1.01 (0.930–1.11)	0.741	1.43 (1.29–1.58)	< 0.001	0.726 (0.648–0.814)	< 0.001
No caste/tribe	1.06 (0.946–1.19)	0.313	1.15 (0.989–1.34)	0.068	0.741 (0.606–0.906)	0.003	0.781 (0.636–0.958)	0.0180
Alcohol consuming								
No	1 (ref)		1 (ref)		1 (ref)		1 (ref)	
Yes	4.54 (4.30–4.80)	<0.001	5.22(4.88-5.59)	< 0.001	4.84 (4.53–5.18)	< 0.001	4.90 (4.55–5.28)	< 0.001
Religion								
Hindu	1 (ref)		1 (ref)		1 (ref)		1 (ref)	
Muslim	1.02 (0.944–1.11)	0.57	1.28 (1.17–1.40)	0.081	0.948 (0.859–1.05)	0.296	1.37 (1.23–1.52)	< 0.001
Others	0.603 (0.554–0.657)	< 0.001	0.529 (0.473–0.592)	0.265	0.687 (0.594–0.794)	< 0.001	0.763 (0.651–0.895)	< 0.001
Household’s wealth index								
Poorest	1 (ref)		1 (ref)		1 (ref)		1 (ref)	
Poorer	0.709 (0.667–0.753)	< 0.001	0.963 (0.896–1.03)	0.302	0.659 (0.609–0.713)	< 0.001	0.917 (0.839–1.00)	0.053
Middle	0.487 (0.459–0.517)	< 0.001	0.789 (0.733–0.849)	< 0.001	0.445 (0.409–0.485)	< 0.001	0.818 (0.735–0.909)	< 0.001
Richer	0.368 (0.344–0.393)	< 0.001	0.664 (0.607–0.727)	< 0.001	0.322 (0.291–0.356)	< 0.001	0.681 (0.598–0.775)	< 0.001
Richest	0.239 (0.221–0.259)	< 0.001	0.482 (0.435–0.534)	< 0.001	0.207 (0.180–0.239)	< 0.001	0.512 (0.428–0.613)	< 0.001
**Spatial factors**								
Place of residence								
Rural	1 (ref)		1 (ref)		1 (ref)		1 (ref)	
Urban	0.680 (0.645–0.719)	< 0.001	1.10 (1.03–1.17)	0.003	0.667 (0.613–0.730)	< 0.001	1.13 (1.02–1.23)	0.016
Region								
South	1 (ref)		1 (ref)		1 (ref)		1 (ref)	
West	1.72 (1.57–1.88)	< 0.001	3.23 (2.91–3.58)	< 0.001	1.71 (1.40–2.09)	< 0.001	2.82 (2.33–3.43)	< 0.001
North-East	4.26 (3.84–4.72)	< 0.001	5.22 (4.57–5.98)	< 0.001	8.02 (6.90–9.34)	< 0.001	8.50 (7.17–10.1)	< 0.001
East	2.75 (2.53–2.99)	< 0.001	3.26 (2.95–3.60)	< 0.001	4.14 (3.58–4.78)	< 0.001	4.11 (3.52–4.80)	< 0.001
Central	2.83 (2.65–3.04)	< 0.001	4.23 (3.89–4.59)	< 0.001	5.15 (4.51–5.88)	< 0.001	6.11 (5.33–7.00)	< 0.001
North	1.36 (1.26–1.46)	< 0.001	2.35 (2.15–2.57)	< 0.001	1.80 (1.55–2.11)	< 0.001	2.72 (2.32–3.19)	< 0.001
**Access to information**								
Reading newspaper or magazine								
Not at all	1 (ref)		1 (ref)		1 (ref)		1 (ref)	
Less than once a week	0.604 (0.566–0.645)	< 0.001	0.913 (0.841–0.990)	0.0274	0.701 (0.634–0.776)	< 0.001	0.978 (0.868–1.10)	0.711
At least once a week	0.468 (0.443–0.494)	< 0.001	0.884 (0.823–0.949)	< 0.001	0.554 (0.513–0.598)	< 0.001	0.975 (0.884–1.08)	0.614
Almost every day	0.323 (0.305–0.342)	< 0.001	0.811 (0.750–0.876)	< 0.001	0.346 (0.317–0.377)	< 0.001	0.903 (0.800–1.02)	0.103
Frequency of watching television								
Not at all	1 (ref)		1 (ref)		1 (ref)		1 (ref)	
Less than once a week	0.929 (0.865–0.997)	0.042	1.11 (1.02–1.20)	0.014	1.05 (0.956–1.16)	0.291	1.23 (1.10–1.37)	0.132
At least once a week	0.731 (0.684–0.781)	< 0.001	1.08 (0.995–1.17)	0.065	0.802 (0.734–0.876)	< 0.001	1.15 (1.04–1.28)	0.527
Almost every day	0.454 (0.429–0.481)	< 0.001	1.06 (0.983–1.14)	0.129	0.447 (0.410–0.487)	< 0.001	1.22 (1.05–1.42)	0.325
Frequency of listening radio								
Not at all	1 (ref)		1 (ref)		1 (ref)		1 (ref)	
Less than once a week	1.07 (0.999–1.15)	0.054	1.09 (1.00–1.18)	0.043	1.33 (1.20–1.47)	< 0.001	1.42 (1.22–1.64)	< 0.001
At least once a week	0.832 (0.779–0.888)	< 0.001	0.955 (0.878–1.04)	0.279	0.980 (0.884–1.09)	0.699	1.28 (1.11–1.47)	0.009
Almost every day	0.678 (0.624–0.737)	< 0.001	0.946 (0.862–1.04)	0.240	0.791 (0.687–0.910)	0.001	1.30 (1.07–1.59)	0.010

^a^Data are from the fourth Indian National Family Health Survey. Unadjusted and adjusted odds ratios with 95% CIs in parentheses are shown. *P* value was displayed in a separate column. All data are weighted to account for survey design

Most of the variables that were associated with any tobacco use significantly were also correlated with both smoked and smokeless tobacco (SLT) use. For example, increasing age, lower education, working in any occupations, higher economic status, drinking alcohol, urban residents, and regions. However, the correlation of different ethnic groups, various religions, and listening radio with both smoked and SLT using became significant. Compared with caste, respondents with the tribe and no caste/tribe had lower odds of dual-use. The adjusted odds ratio of both smoked and SLT use was 1.13 times in the urban area. Men from Muslim religions were more likely (AOR = 1.37; 95% CI 1.23–1.52) being dual users while opposite situations were found among men from other religions (AOR = 0.763; 95% CI 0.651–0.895).

## Discussion

This study gives a comprehensive picture of the prevalence and determinants of tobacco use among Indian men aged 15–54 years using a recent national survey. Almost one in every two men (45.5%) used tobacco in 2015–2016 in India. Age, lower education, occupation, region, alcohol consumption, separated/divorced/widowed men, and richest economic status substantially had a stable association with all four groups of tobacco use described in this study. The magnitude and correlation of other selected variables differ according to the forms of tobacco use described below. The findings of the studies from India [9], Nepal [18], Afghanistan [19], and Ethiopia [20] are coherent with the finding of our study.

### Prevalence of different forms of tobacco use

Around one-fourth of men use smoking (24.6%) and one-third used SLT (29.1%) while one in every ten men used both smoked and SLT (8.4%). The prevalence of all groups of tobacco use is decreasing in India in the last two decades [11, 13]. The prevalence of smoking (27.3%), SLT (40.2%), and any form of tobacco (52.3%) was higher in the neighboring country, Nepal [18]. Our study found that the prevalence of smoking was lower than the proportion of SLT use. The result is consistent with the finding of other studies conducted in India [13, 21].

Tobacco use was more prevalent among men in the older group, lower wealth status and education, manual occupation, separated/divorced/widowed, alcohol consumer, residents of the rural area and north-east region, and less access to information. Studies conducted in India found that increasing age, living in rural areas, low education, and economic status increased tobacco use [11, 13, 22]. Khanal et al. [22] also found that tobacco use was more prevalent among men who have less access to information and have manual working status. The residence of the north-east region was more prevalent to being a tobacco user also as reported by another Indian study [13]. Smoking was more common among respondents from Muslim religion and ethnic groups having no caste/tribe. The finding is coherent with an Indian study [11].

### Factors associated with tobacco use

The strength of the association of consuming alcohol and residing in the north-east region with tobacco use was stronger than other variables. The magnitude of alcohol consumption was stronger for smoking than smokeless tobacco use. Tang et al. [23] also showed a similar finding in Ethiopia. People who consume alcohol more tend to smoke more [15]. Similarly, smokers have 2.7 times more risk to be alcohol users than men who do not smoke. Control programs of alcohol abuse should not be isolated from the tobacco control program [24]. A higher association of tobacco use with the north-east region is due to the significant effects of peers and cultural acceptance of tobacco using in the north-east region [25].

The men who were engaged in manual work were more prone to be a tobacco user. High working hours and working conditions would be a possible cause for more tobacco use among this group [26]. On the other hand, people who engaged in professional work tend to avoid tobacco use in some office settings [15]. Increasing age positively correlated with smoking, any tobacco, and dual-use. Similar findings were reported by studies from India [15] and Ethiopia [27, 28]. The longer period for the trial of tobacco consumption is one reason for higher users among elders [28].

Higher education and economic status had a strong protective effect on tobacco use. Recent studies in India also reported that higher education and wealth status correlated with tobacco use [9, 12]. Highly educated men usually have better self-efficacy, healthy behavior, and high access to information [15]. Men with poor wealth quintiles have a lack of awareness of tobacco hazards, and the economic burdens and stress trigger them to use tobacco [29]. Additionally, respondents who are separated/divorced/widowed during the study period were more prone to be a tobacco user. Our findings are consistent with a study performed in Ethiopia [20, 22].

Ethnicity, religion, and the living urban area had a significant association with smoking [20]. Men from no caste/tribe and other religious groups were less likely SLT users whereas the association of tribe ethnic group, Muslim religion, and living in urban with SLT use were not significant. Living in urban was not associated with SLT in Nepal [22] and in India [11].

In access to information, watching television and listening to radio associated with smoking while reading newspaper had an insignificant correlation. A similar result was demonstrated by a recent study based on a national representative study in Afghanistan [19]. Reading newspapers and listening to the radio at least once a week and almost every day had a weaker protective effect on using any tobacco and SLT while the correlation of watching television with SLT use was found insignificant. A study conducted in Nepal reported reading newspapers or magazines protect from being SLT users [22]. Specific health messages should be disseminated through specific media as the finding shows that association varied between forms of tobacco use and type of media.

### Public health implication

The burden of tobacco use is a great public health problem in India. Policymakers need to develop innovative and cost-effective strategies to mitigate the burden of tobacco use. One important policy implication of our findings is that the high-risk group, the men from lower wealth status, less education, north-east region, and manual working status should be targeted to reduce tobacco use. Increasing the Social Behavior Change Communication (SBCC) and awareness campaigns about the danger of tobacco use needs to be implemented aiming to spread messages and being behavior change among tobacco users. All types of national, local, and social media should be used to disseminate the messages. As alcohol consumption triggers tobacco use, anti-smoking campaigns should also focus on reducing alcohol abuse. Finally, long-term success in curbing the burden of smoking will require political commitment including harmonized legal provisions, such as reducing tobacco marketing, formal education about the dangers of tobacco use, progressive tax, packaging, and labeling of tobacco products and price strategies.

### Strengths and limitations

We analyzed nationally representative data with a high response rate. The results of this study are generalizable. We applied sample weight, cluster effect, and complex sampling during our analysis, and collected 95% CI with point estimates. These actions increase the precision of the study findings. The main limitation of this study is the survey, NFHS-4, which is focused on maternal and child health and reproductive health in women, and the target population was limited to aged 15–54 years in men. Our finding shows the prevalence and association of tobacco use in men increase with age. The exclusion of elderly men may affect the finding we found in this study. The prevalence data collected by self-reporting generally underestimated as tobacco using sometimes correlated with a social stigma. The cross-sectional design of this study limits from drawing causal inferences.

## Conclusions

Our study showed one in every two Indian men was consuming tobacco in 2016. We identified lower economic and education levels, elderly, manual working status, residence in the north-east region, and alcohol consumption were the important determinants of tobacco use among men aged 15-54 years in India. Innovative strategies targeting high-risk groups are crucial to curbing tobacco consumption in India.

## Data Availability

Data for this study are available through the MEASURE Evaluation Data verse. The dataset of this study is available through the DHS website (https://dhsprogram.com/what-we-do/survey/survey-display-355.cfm).

## Abbreviations

AOR: Adjusted odds ratio
CI: Confidence interval
GDP: Gross domestic product
NFHS: National Family Health Survey
NCDs: Non-communicable diseases
SLT: Smokeless tobacco
SIDS: Sudden infant death syndrome

## References

[1] Alebshehy R (2019). WHO report on the global tobacco epidemic. 2019.

[2] Reddy KS, ath; C. Gupta P. Tobacco controls in India [Internet]. New Delhi, India; 2004. Available from: https://nhm.gov.in/NTCP/Surveys-Reports-Publications/Report_on_Tobacco_Control_in_India.pdf.

[3] Maritz GS, Mutemwa M (2012). Tobacco smoking: patterns, health consequences for adults, and the long-term health of the offspring. Glob J Health Sci..

[4] US Department of Health and Human Services. The health consequences of smoking—50 years of progress: a report of the surgeon general. Atlanta, GA: US Department of Health and Human Services, Centers for Disease Control; 2014.

[5] Goodchild M, Nargis N, d’Espaignet ET (2018). Global economic cost of smoking-attributable diseases. Tob Control..

[6] Gupta, PC; Ray C. Epidemic in India. In: Boyle P, Gray N, Henningfield J, Seffrin J, Zatonski W (eds). Tobacco: science, policy and public health. Oxford; 2004.

[7] Pednekar MS, Vasa J, Narake SS, Sinha DN, Gupta PC (2016). Tobacco and alcohol associated mortality among men by socioeconomic status in India. Epidemiol Open J..

[8] Ministry of Health and Family Welfare. Economic burden of tobacco related diseases in India-executive summary. 2011.

[9] Pradhan MR, Patel SK, Prusty RK (2019). Pattern and predictors of tobacco use in India: evidence from National Family Health Survey (2015–2016). J Health Manag..

[10] Viswanath K, Ackerson LK, Sorensen G, Gupta PC (2010). Movies and TV influence tobacco use in India: findings from a national survey. PLoS One..

[11] Rani M, Bonu S, Jha P, Nguyen SN, Jamjoum L (2003). Tobacco use in India: prevalence and predictors of smoking and chewing in a national cross sectional household survey. Tob Control..

[12] Singh PK, Yadav A, Singh L, Singh S, Mehrotra R. Social determinants of dual tobacco use in India: an analysis based on the two rounds of global adult tobacco survey. Prev Med Reports. 2020:101073. 10.1016/j.pmedr.2020.101073 PMC7125349 32257776

[13] Singh A, Ladusingh L (2014). Prevalence and determinants of tobacco use in India: evidence from recent Global Adult Tobacco Survey data. PLoS One..

[14] Kahar P, Misra R, Patel TG. Sociodemographic correlates of tobacco consumption in rural Gujarat, India. Pednekar MS, editor. Biomed Res Int [Internet]. 2016;2016:5856740. Available from: 10.1155/2016/5856740. 10.1155/2016/5856740 PMC4834402 27127788

[15] Manimunda SP, Benegal V, Sugunan AP, Jeemon P, Balakrishna N, Thennarusu K (2012). Tobacco use and nicotine dependency in a cross-sectional representative sample of 18,018 individuals in Andaman and Nicobar Islands, India. BMC Public Health..

[16] International Institute for Population Sciences. National Family Health Survey (NFHS-4), 2015-16: India. Mumbai:: International Institute for Population Science and ICF. 2017.

[17] Wikipedia Contributors . Administrative divisions of India [Internet]. Wikipedia, The Free Encyclopedia. 2019.

[18] Das GR, Jahan M, Hasan M, Sutradhar I, Sajal IH, Haider SS, et al. Factors associated with tobacco use among Nepalese men aged 15–49 years: data from Nepal demographic and Health Survey 2016. Clin Epidemiol Glob Heal. 2020.

[19] Alemi Q, Stempel C, Montgomery S. Prevalence and social determinants of tobacco use in Afghanistan. Int Health. 2020. 10.1093/inthealth/ihaa010 PMC7807235 32304214

[20] Guliani H, Gamtessa S, Çule M (2019). Factors affecting tobacco smoking in Ethiopia: evidence from the demographic and health surveys. BMC Public Health..

[21] Ministry of Health and Family Welfare G of I. Global Adult Tobacco Survey-[Fact Sheet] [Internet]. New Delhi; 2012. Available from: http://www.who.int/tobacco/surveillance/survey/gats/GATS_India_2016-17_%0AFactSheet.pdf.

[22] Khanal V, Adhikari M, Karki S (2013). Social determinants of tobacco consumption among Nepalese men: findings from Nepal Demographic and Health Survey 2011. Harm Reduct J..

[23] Tang S, Bishwajit G, Luba TR, Yaya S (2018). Prevalence of smoking among men in Ethiopia and Kenya: a cross-sectional study. Int J Environ Res Public Health..

[24] Breslau N (1995). Psychiatric comorbidity of smoking and nicotine dependence. Behav Genet..

[25] Ladusingh L, Dhillon P, Narzary PK. Why do the youths in Northeast India use tobacco? J Environ Public Health. 2017. 10.1155/2017/1391253 PMC5470014 28642795

[26] Cho Y-S, Kim H-R, Myong J-P, Kim HW (2013). Association between work conditions and smoking in South Korea. Saf Health Work..

[27] Reda AA, Moges A, Yazew B, Biadgilign S (2012). Determinants of cigarette smoking among school adolescents in eastern Ethiopia: a cross-sectional study. Harm Reduct J..

[28] Rudatsikira E, Abdo A, Muula AS (2007). Prevalence and determinants of adolescent tobacco smoking in Addis Ababa, Ethiopia. BMC Public Health..

[29] Kassim S, Rogers N, Leach K (2014). The likelihood of khat chewing serving as a neglected and reverse ‘gateway’ to tobacco use among UK adult male khat chewers: a cross sectional study. BMC Public Health..

